# Assessment instrument of risk factors for exposure to SARS-CoV-2 among health care workers with suspected COVID-19

**DOI:** 10.47626/1679-4435-2022-717

**Published:** 2022-03-30

**Authors:** Marcia Cristina Bandini, Sérgio Roberto de Lucca, Priscila Bergamo Francisco, Patrícia Falabella Leme

**Affiliations:** 1 Área de Saúde do Trabalhador, Departamento de Saúde Coletiva, Faculdade de Ciências Médicas, Universidade Estadual de Campinas (Unicamp), Campinas, SP, Brazil.; 2 Área de Epidemiologia, Departamento de Saúde Coletiva, Faculdade de Ciências Médicas, Unicamp, Campinas, SP, Brazil.; 3 Centro de Saúde da Comunidade, Unicamp, Campinas, SP, Brazil.

**Keywords:** occupational health, coronavirus 2, risk assessment, health professionals

## Abstract

**Introduction:**

Health professionals are at high or very high risk of being infected with COVID-19, making it essential to adopt control and protection measures. The World Health Organization developed a risk assessment instrument to identify possible protection failures and to guide corrective actions.

**Objectives:**

To test an adapted version of this instrument among health care workers with a suspected case of COVID-19.

**Methods:**

The World Health Organization instrument was translated and adapted with the participation of health care workers. The adapted version was inserted into Google Forms^®^ and applied to 211 health care workers with a suspected case of COVID-19 in three public hospitals.

**Results:**

Fifty-five percent of workers were nursing professionals. The main risk factors for exposure to the severe acute respiratory syndrome coronavirus 2 (SARS-CoV-2) were failures in training, in donning and doffing, and in the use and maintenance of personal and collective protective equipment; problems in cleaning rooms and equipment; and changes in work organization. The assessment instrument fulfilled its purpose of assessing risk factors for exposure to SARS-CoV-2 and helped adopt the corrective and preventive measures required to prevent COVID-19 among health care workers.

**Conclusions:**

The adapted instrument proved to be an important support tool to improve risk management and protection of these workers.

## Introduction

COVID-19 is a disease caused by the severe acute respiratory syndrome coronavirus 2 (SARS-CoV-2),^1^ whose transmission occurs through several forms, from a sick person to another or through contact with secretions and/or excretions contaminated with the virus through handshake, droplets of saliva, sneezing, coughing, catarrh, contaminated objects or surfaces.^2^

Due to the lack of specific treatments and to ongoing vaccination, this community-transmitted disease requires sanitary barriers, such as social isolation,^3^ generating financial impacts both for individuals and for several economic sectors.^4^ Therefore, work plays a major role in the advance of cases of COVID-19, since many people are obliged to go out to work due to employer’s request or to economic need.^5^

Public policies have an influence on the behavior of COVID-19. In Brazil, the number of essential activities, considered “indispensable to meet community needs,” has increased since the beginning of the pandemic, including workers involved in health care, cleaning services, postal service, and urban waste collection, as well as caregivers, police officers, soldiers, firefighters, postmen, drivers, and application delivery people, among others.^6^ This increase - associated with decreased rates of social isolation in many municipalities^7^ - made the disease spread rapidly: Brazil is the country with the 2nd highest number of cases in the world,^8^ leading to overloaded health services and increased risk of contamination among workers directly involved in COVID-19 care.

Considering professional activities, the United States of America proposed four levels of risk of occupational exposure to SARS-CoV-2: very high, high, medium, and lower risk. Such characterization depends on professional activity, on the likelihood of exposure to the biological agent when performing certain procedures, and on the contact with the general public, representing the likelihood of being contaminated at work.^9^ Health professionals belong to the high or very risk group; thus, it is essential to adopt control and protection measures, both to preserve the health workforce and to prevent contamination of patients hospitalized for diseases other than COVID-19.

According to the World Health Organization (WHO), employers and managers at health institutions should ensure that all preventative and protective measures are taken to minimize the risks of contamination to which workers are exposed in the COVID-19 pandemic. Appropriate risk management meets the technical and ethical goal of protecting health professionals. Among the measures recommended by the WHO, the most important are provision of personal protective equipment (PPE), appropriate training, based on protocols and procedures for the safe performance of activities, consultation and participation of workers in the process, and guarantee of work leave in situations of danger and emerging risk or suspected contamination.^10^

In order to study the efficacy of protective measures and to assess the exposure risk of health care workers, the WHO published a risk assessment instrument that contributed to guide managers on possible process failures, where and how they occurred, and what corrective actions are necessary to eliminate or mitigate risks.^11^ This study aimed to test an adapted version of this instrument among health care workers with suspected COVID-19 by applying it in three public health services in the state of São Paulo, Brazil.

## Methods

The research instrument was developed by a committee of Occupational Health experts on the basis the WHO Risk Assessment tool published on March 19, 2020, loosely translated into Portuguese. Experts included new questions to the translated version, considering their own experience and field observations that were conducted by one of the researchers in two of the three hospitals selected for the study. The second version was tested through interviews with health care workers, and results were discussed with the expert committee. A third version was then applied in the study, which used the Google Forms^®^ platform for data collection.

The target population of the study consisted of health workers, not exclusively health care professionals, who worked at three public hospitals located in a municipality in the state of São Paulo and who attended the Community Health Center maintained by the university. Inclusion criteria considered workers potentially exposed to SARS-CoV-2 who presented with flu-like syndrome, defined as acute respiratory illness characterized by feverish sensation or fever, even if self-reported, together with coughing, sore throat, running nose, or breathing difficulty,^12^ and who were treated during the 30-day study period. Participation in the study was voluntary. All workers who attended the Community Health Center during the research period were invited to participate.

Only participants who agreed with and signed the Informed Consent Form (ICF) on the application had access to the online questionnaire. The study was approved by the Research Ethics Committee under no. CAAE 31585820.2.0000.5404.

## Results

### Research instrument

The version translated and expanded by the expert committee was tested with 50 health care workers through interviews. The results were rediscussed with the committee, which developed the third version, consisting of 11 sections, 10 with closed questions (Table 1) and one last section with an open question for workers to leave their comments.

**Table 1:** Assessment questionnaire of COVID-19 risk for health institutions

Section	Question	Response options
ICF	Informed Consent Form (ICF). Do you agree to participate?	Yes; No
Personal information	Name:	Descriptive
	Date of birth:	Descriptive
	Sex:	Female; Male
	Estimated monthly family income in BRL (considering income from all family members):	Descriptive (numerical); I prefer not to answer
	Your educational level:	Incomplete elementary education; Complete elementary education; Complete high school; Complete higher education
	Of the people who live with you, how many are older than 60 years of age?	Descriptive (numerical)
	Of the people who live with you, how many are younger than 60 years of age?	Descriptive (numerical)
	Does any household member have chronic diseases (hypertension, diabetes, asthma)?	Yes; No If yes, please describe who and what disease
	Does your house have basic sanitation?	Yes; No
	Number of bathrooms in the house:	Descriptive (numerical)
	Contact information:	Descriptive (telephone and e-mail)
Professional information	Registration number/ID:	Descriptive (numerical)
	Place of work:	Hospital 1; Hospital 2; Hospital 3
	Hospital unit:	Descriptive
	Occupation/position:	Descriptive
	Summary of activities performed at work:	Descriptive
	Average weekly working hours at this hospital, in hours:	Descriptive (numerical)
	Do you have another job beside this one?	Yes; No; I prefer not to answer
	If yes, are you exposed to COVID-19 in this other job?	Yes; No; Not applicable
	Average weekly working hours in the other job, in hours:	Descriptive (numerical); I prefer not to answer
Information on your current health	(A) Current symptoms:	Fever; dyspnea (shortness of breath); cough; sore throat; muscle pain; loss of smell; headache; other (please describe)
	Date of symptom onset	Descriptive
	(B) Indicate whether you have any of the following health problems:	Chronic respiratory diseases (asthma, bronchitis, emphysema); chronic heart diseases; diabetes; chronic kidney diseases; immunological diseases or treatment with immunosuppressive drugs; genetic/chromosomal diseases; high-risk pregnancy; arterial hypertension; other (please describe)
Potential community exposure (last 30 days)	(A) Recently, has someone you live with had COVID-19?	Yes; No; Do not know
	(B) Recently, have you had contact with someone who has/had COVID-19 at places such as school, church, market, or other?	Yes; No; Do not know
	(C) Do you often use public transportation (bus, train)?	Yes; No
Potential occupational exposure (last 30 days)	(A) Have you had direct contact with a patient with confirmed COVID-19?	Yes; No; Do not know
	(B) Have you had close contact (e.g., less than 1 meter, but with no direct contact) with a patient with confirmed COVID-19?	Yes; No; Do not know
	(C) Have you participated in any aerosol-generating procedure or have been present in the same environment?	Yes; No; Do not know
	If yes, what type?	Tracheal intubation; nebulizer use; airway aspiration; sputum collection; tracheostomy; bronchoscopy; other (please describe)
	At work, have you had direct contact with a site where a patient with confirmed COVID-19 was treated? (e.g.: hospital bed, restroom, medical equipment used in treatment, ambulance, others)	Yes; No; Do not know
Protective measures during work at Hospitals 1, 2 or 3:	(A) Have you received instruction about personal protective equipment (PPE) that you should use in each work situation?	Yes; No; Do not know
	If yes, in what format?	Written instruction; face-to-face theoretical training; face-to-face training with observed practice; video training; other (please describe)
	If yes, how do you assess the training received?	Very effective; Little effective; Not effective
	(B) Have you received the PPE that you use in each work situation?	Yes; No; Not applicable
	(C) Indicate what type of PPE you use:	Surgical mask; N95 mask (“duckbill”); disposable gown; safety goggles; face shield; gloves; cap
	(D) Do you reuse any of the PPE?	Yes; No; Not applicable
	If yes, which type?	Descriptive
	(E) In case of reuse, where is your PPE (e.g.: N95 mask) stored until the next use?	Descriptive
	(F) Is your donning performed with the assistance of other professional?	Yes; No; Not applicable
	(G) Is your PPE doffing performed with the assistance of other professional?	Yes; No; Not applicable
	(H) Are donning and doffing performed in different places?	Yes; No; Not applicable
	(I) Are goggles and face shields sanitized in a place intended for this purpose, with no risk of contamination with other PPE?	Yes; No; Not applicable
	(J) Do you take PPE home?	Yes; No; Not applicable
	If yes, which one?	Descriptive
	If you work at other place beside this hospital, is there any difference in protective measures? If yes, which is it?	Yes; No; Not applicable
For the next questions, please quantify the frequency with which you have been using PPE, according to the recommendations received. Please select “always” if you use PPE more 95% of times recommended; “most of the time” if you use PPE from 50 to 95% of times recommended; “occasionally” if you use PPE from 20 to 50% of times recommended; and “rarely” if you use PPE less than 20% of times recommended.		
Protective measures during work at Hospital 1, 2 or 3:	(K) During care to a patient with COVID-19 (suspected or confirmed), how often do you use the following PPE, according to the instructions for each specific procedure? - Gloves - Mask (surgical or N95) - Face shield/safety goggles - Disposable gown - Cap	Always; Most of the time; Occasionally; Rarely; Not applicable
	(L) Do you use PPE according to the protocol for which you were trained, replacing the items when necessary?	Always; Most of the time; Occasionally; Rarely; Not applicable
	(M) Do you sanitize your hands with soap and water, alcohol 70%, or another recommended product before AND after the following situations involving patients with COVID-19? - Touching a patient - In clean procedures (e.g..: intubation, passing catheter, others) - In procedures with exposure to blood, urine, feces - After touching surfaces close the patient (e.g.: doors, beds, remote controls)	Always; Most of the time; Occasionally; Rarely; Not applicable
Environmental precautions	(A) In environments where you work and where there is greater risk of interaction with patients with COVID-19, are surfaces cleaned at least 3 (three) times a day or after providing care to a confirmed case of COVID-19?	Always; Most of the time; Occasionally; Rarely; Not applicable
Accidents with exposure to biological material	(A) In the last 30 (thirty) days, have you had any occupational accident with exposure to blood or respiratory secretions (e.g.: accidents with sharps objects; droplets on eyes, mouth, nose, and/or non-intact skin)?	Yes; No; Do not know
Work organization in the context of COVID-19	(A) In situations of greater risk of contamination with COVID-19, do you ask help to a coworker?	Yes; No; Do not know
	(B) Do you help a coworker who asks for help in a situation of greater risk of contamination with COVID-19?	Yes; No; Do not know
	(C) Have you started to perform activities that you did not usually do before? (ex.: protective measure, patient care tasks, cleaning procedures, disinfection/disposal of material)?	Yes; No; Do not know
	(D) Have there been changes in the routine that required learning new things and implied in changes in contamination risk?	Yes; No; Do not know
	(E) Have there been changes in patient flows, division of tasks, situations of work overload (increased working hours, increase in the number of patients to be treated)?	Yes; No; Do not know
	(F) Has there been a decrease in the number of professionals in the staff?	Yes; No; Do not know
	(G) Has there been shortage of work material?	Yes; No; Do not know
	(H) Do you feel that your autonomy to decide has been affected?	Yes; No; Do not know
Final comments	(A) Would you like to add something?	Descriptive

Adapted version of the World Health Organization Risk Assessment, in its version of March 19, 2020.

### Assessment of risk factors for COVID-19

During the research period, 211 participants answered the questionnaire, most of whom were women (82.9%), with a mean age of 38 years, were white (69.6%), and had complete higher education (57.3%). Furthermore, 36.5% of workers reported having some chronic disease, the most cited of which were arterial hypertension (24 workers), respiratory diseases (21), thyroid diseases (13), diabetes mellitus (9), and heart diseases (10).

With regard to housing conditions, 35% reported living with at least one person with a non-communicable disease in the same household.

In terms of employment relationship, 63% of workers were regular employees, 20% were statutory employees, 14% were medical residents, and 6% had other types of employment relationship with the institution. As for occupation, nursing technicians and assistants were the most representative group, accounting for 40% of participants, followed by nurses (15%), residents (11%), physicians (9%), administrative workers (6%), the pantry/kitchen workers (3%), and cleaners (2%). Other occupations accounted for 30% of the sample, but they mentioned by less than 1% of respondents each.

Half participants (50%) reported having direct contact with COVID-19 patients in their work activities, 35% reported not having direct contact with confirmed cases, and 15% did not know. Among those who reported direct contact, 36% routinely performed aerosol-generating procedures. The regular use of PPE was confirmed by almost all workers (92%), although 6% still reported difficulties in its use. With regard to reuse or prolonged use, most respondents (79%) stated to reuse PPE, especially PPF2 masks and face shields, and 23% told that they took PPE home.

Training failures were identified: 10% of participants reported not receiving training on PPE, and 25% of those who received training considered it little effective (23%) or no effective (2%). Failures related to risk of cross-contamination were also found. Dunning and doffing were performed without assistance in 58% and 63% of the cases, respectively, and 25% of workers reported to perform dunning and doffing in the same place, which increases the likelihood of cross-contamination. Sanitation of some PPE items (safety goggles and/or face shields) was performed in the same place where dunning was performed in 20% of the cases.

With regard to environmental cleaning, only 49% of participants reported that it was performed at least three times a day - as recommended by good practices. In terms of availability of material (PPE, routine activities), 26% reported lack or shortage, especially in the first months of the pandemic.

Relevant changes were identified in work organization: 80% of participants said they started to perform new activities in the context of COVID-19 pandemic, and 86% reported that new routines required learning new things. Moreover 36% believed that their autonomy in decision-making was affected. Work overload was reported by 80% of participants, including reduced staff (67%). Only 10% reported not asking for help even in higher risk situations, and 4% said they did not help coworkers in a similar situation.

In the open-ended question, the most relevant comments, according to researchers’ critical analysis, were: (1) the need for standardizing the level of protection between COVID and non-COVID care areas; (2) request for testing all health care workers, regardless of presence of suspected symptoms of COVID-19; (3) reinforcement and improvement of training, especially for aerosol-generating procedures; (4) possibility of greater workers’ participation in decision-making and in the development of the plan to fight against COVID-19 in the analyzed institutions.

## Discussion

In this study, the sociodemographic profile of workers potentially exposed to SARS-CoV-2 and with suspected COVID-19 who attended the Community Health Center during the study period reveals a relatively young (mean age 38 years) and predominantly female (82.9%) workforce, of which 36.5% reported having at least one chronic disease. These characteristics are consistent with those of a study conducted by the Centers for Disease Control and Prevention (CDC) on the profile of health professionals with COVID-19, which found that most of these professionals are female (73%), with a mean age of 42 years, and 38% have some type of underlying chronic condition.^13^

According to the CDC, the presence of certain health conditions increases the risk of developing the most severe forms of COVID-19, including the most cited conditions in our study (arterial hypertension, respiratory diseases, diabetes mellitus).^14^ Therefore, it is noteworthy that one third of workers remained exposed to SARS-CoV-2, which may be explained by the staff reduction reported by 67% of study participants.

In addition to the increase in the risk of workers themselves, household transmission, with the development of more severe cases, is a source of concern for 35% of respondents, who reported living with at least one person with a non-communicable chronic disease in the same household. Therefore, either by direct exposure or by the possibility of carrying the virus to their own household, the work of these professionals shows to be a determining factor in the spread of COVID-19 in the community where they live and work. This risk is increased by the fact that many workers (23%) reported taking potentially contaminated PPE home.

More than a half respondents (55%) worked in the nursing field, considering nurses (15%) and nursing technicians and assistants (40%). The contamination rate of nursing professionals is estimated to be three times as high as that of physicians.^15^ Thus, appropriate assessment of risk factors is particularly important for these workers, especially in the training on appropriate use of PPE and on donning and doffing, as well as on precautions in aerosol-generating procedures, such as endotracheal intubation, bronchoscopy, open aspiration, nebulizer treatment, and manual ventilation before intubation.

According to Almeida,^16^ “it has been discussed whether the emphasis given on the use PPE, social etiquette, and hygiene measures may have potentially minimized the importance of engineering control and administrative control measures to prevent the disease. Emphasis is given on the need for workers to receive training to acknowledge risk situations associated with difficulties in the new activities arising from the pandemic, focusing on new interactions between coworkers and between workers, as well as on new instruments and contexts resulting from the pandemic”.

In this sense, the present study showed that, although the use of PPE was confirmed by the vast majority of respondents (92%), several protection failures were identified in relation to WHO recommendations to respond to the COVID-19 pandemic^17^ or to the Brazilian recommendations developed by the Ministry of Health.^18^

PPE acts as a protection barrier and may prevent biological contamination, but its efficacy depends on training on proper donning and doffing procedures, on hygiene conditions in use, reuse, and prolonged use, and on appropriate planning of care flow so as to prevent cross-contamination. Doffing is particularly critical, because it is performed after the conclusion of patient care, which facilitates for workers to “let down their guard”.^15^ This study made it possible identify some important failures in preventing contamination with SARS-CoV-2 among health care workers, such as training, considered little or no effective by 25% of respondents, and unassisted donning and doffing, mentioned by 58% and 63% of them, respectively, in addition to the possibility of cross-contamination when donning and doffing are performed in the same place, which occurred in 25% of reports, or when used PPE is sanitized in the same place where donning is performed, which occurred in 20% of the cases.

Respiratory etiquette measures, hand washing, and procedures of environmental and material hygiene are relevant to prevent COVID-19, especially in settings that treat patients with this disease. The presence of the virus on surfaces, door handles, keyboards, and other objects in the hospital environment has been the object of study, in with the purpose of understanding the mechanism of disease transmission. A study conducted in a hospital in Singapore that analyzed 245 surface samples from three isolation rooms for COVID-19 and 27 general wards found that 56.7% of surfaces were contaminated with SARS-CoV-2.^19^ Therefore, it is important to identify the quality and frequency of hygiene measures in workplaces, preferably from the perception of workers themselves. In the case of our study, it was found that only half (49%) of them reported that workplaces were duly sanitized, three times a day.

The difference in the protection levels between workers who act in the so-called COVID vs. non-COVID areas and the non-availability of tests for asymptomatic professionals, especially at the beginning of the pandemic, were important complaints identified in the study. It is currently known that transmission may occur from unacknowledged sources and/or from contact with pre-symptomatic or asymptomatic individuals.^20^ In this study, 11% of workers were not health professionals, despite working at the service; among them, there are cleaners (2%), pantry/kitchen workers (3%), and administrative workers (6%). In this sense, the spaces where workers can interact during working hours - such as pantries, cafeterias, and changing rooms - should receive special attention, because they are often small and unventilated spaces where precautionary measures are likely to be neglected. We recommend, whenever possible, not limiting testing of health care workers to symptomatic individuals or to screening of contacts after acknowledged occupational exposures, since there is the risk of not identifying possible spreaders. Instead, we recommend expanding testing to all asymptomatic individuals and to symptomatic ones identified through screening for fever and respiratory symptoms at the beginning of their shifts.

Health care workers are on the frontline in the fight against the COVID-19 pandemic, being exposed to biological agents, long working hours, stress, fatigue, burnout, stigma, and physical and psychological violence.^21^ In this context, human failures may occur, contributing to increase the risk of biological contamination, and changes in work organization are particularly important and are worsened by the absence of coworkers who got sick. Work overload and new tasks and routines were reported by participants, and one third of them felt that their autonomy was affected. The scenario of fighting against COVID-19 requires workers’ participation in the decision-making process, as shown in the spontaneous reports identified in this study.

## Conclusions

In the scenario of COVID-19 pandemic, the adapted instrument proved to be an important support tool to improve risk management and workers’ protection. It fulfilled the purpose of appointing failures in training and in the proper use and maintenance of PPE, in addition to identifying additional risks related to significant changes in work organization. Therefore, this instrument may be used in different health services, enabling for a more qualified listening of the experiences of workers who work on the frontline in COVID-19 care. Authors recommend expanding the use of the instrument and sharing the results of its application so that it can be improved.
